# Combining β-Carotene with 5-FU via Polymeric Nanoparticles as a Novel Therapeutic Strategy to Overcome uL3-Mediated Chemoresistance in p53-Deleted Colorectal Cancer Cells

**DOI:** 10.1021/acs.molpharmaceut.2c00876

**Published:** 2023-03-28

**Authors:** Pietro Carotenuto, Annalisa Pecoraro, Chiara Brignola, Anna Barbato, Brunella Franco, Giuseppe Longobardi, Claudia Conte, Fabiana Quaglia, Giulia Russo, Annapina Russo

**Affiliations:** † TIGEM, Telethon Institute of Genetics and Medicine, 80078 Naples, Italy; ‡ Medical Genetics, Department of Translational Medical Science, University of Naples “Federico II”, 80131 Naples, Italy; § Department of Pharmacy, University of Naples “Federico II”, 80131 Naples, Italy; ∥ Scuola Superiore Meridionale, School for Advanced Studies, 80138 Naples, Italy

**Keywords:** colorectal cancer, ribosomal protein uL3, β-carotene, chemoresistance, nanoparticles

## Abstract

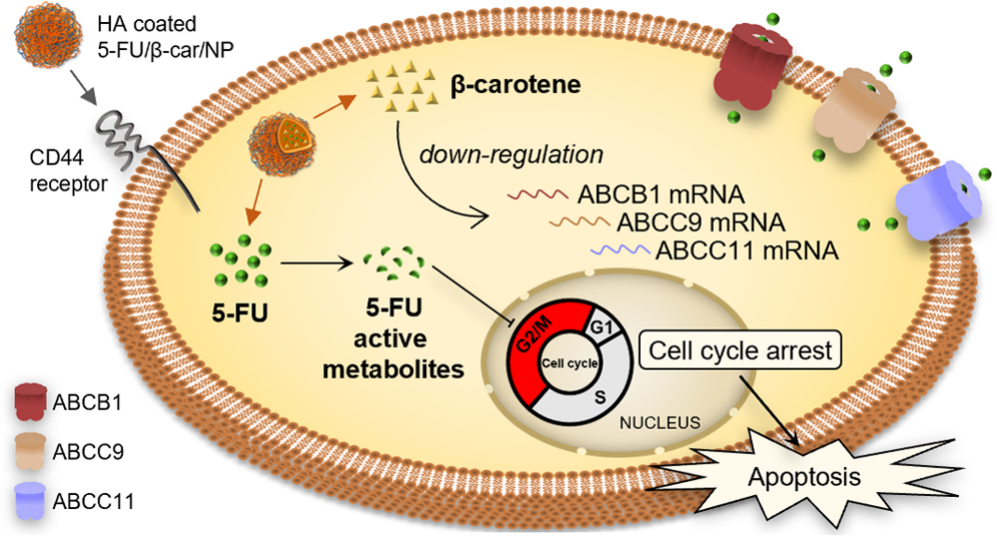

Colorectal cancer (CRC) is one of the leading causes of cancer-related death worldwide. Despite recent therapeutic advancements, resistance to 5-fluorouracil (5-FU) remains a major obstacle to the successful treatment of this disease. We have previously identified the ribosomal protein uL3 as a key player in the cell response to 5-FU, and loss of uL3 is associated with 5-FU chemoresistance. Natural products, like carotenoids, have shown the ability to enhance cancer cell response to drugs and may provide a safer choice to defeat chemoresistance in cancer. Transcriptome analysis of a cohort of 594 colorectal patients revealed a correlation between uL3 expression and both progression-free survival and response to treatment. RNA-Seq data from uL3-silenced CRC cells demonstrated that a low uL3 transcriptional state was associated with an increased expression of specific *ATP-binding cassette* (*ABC*) genes. Using two-dimensional (2D) and three-dimensional (3D) models of 5-FU-resistant CRC cells stably silenced for uL3, we investigated the effect of a novel therapeutic strategy by combining β-carotene and 5-FU using nanoparticles (NPs) as a drug delivery system. Our results indicated that the combined treatment might overcome 5-FU chemoresistance, inducing cell cycle arrest in the G2/M phase and apoptosis. Furthermore, the combined treatment significantly reduced the expression levels of analyzed *ABC* genes. In conclusion, our findings suggest that β-carotene combined with 5-FU may be a more effective therapeutic approach for treating CRC cells with low levels of uL3.

## 1 Introduction

Despite progress in reducing the incidence and mortality rate, colorectal cancer (CRC) is one of the most common and fatal cancers showing multiple genetic mutations and changes.^1,2^ Identifying CRC-related genes represents a key challenge in effective cancer diagnosis and treatment. Among these genes, the tumor suppressor *TP53* has been involved in CRC progression. Mutations or deletions of *TP53* occur for more than half of all CRCs, particularly in patients at the more advanced stages.^3^ Upon abnormal p53 expression during tumor progression, other p53-related genes as those coding for some ribosomal proteins (RPs) are also out of control and have been identified as cancer-related molecules.^4,5^

Chemotherapy is the common treatment approach for CRC, and 5-fluorouracil (5-FU) remains the gold standard of first-line treatment.^6^ 5-FU inhibits DNA synthesis and RNA processing, affecting cell proliferation and survival.^6,7^ It has been demonstrated that some RPs exert several extra-ribosomal functions and are critical players in the 5-FU treatment of cancer cells.^8,9^ Specifically, 5-FU triggers nucleolar stress and consequently induces the release of some RPs from the ribosome to activate p53 by inhibiting the MDM2 pathway. Furthermore, in the absence of p53, 5-FU activates an RP-dependent molecular pathway.^10,11^ In particular, we have previously demonstrated that human ribosomal protein uL3 acts as a stress-sensing molecule and is essential for cancer cell response to 5-FU-induced nucleolar stress.^12,13^

One of the major obstacles to the clinical use of 5-FU is the acquired multidrug resistance (MDR) that frequently occurs during treatment.^14,15^ The identification of novel combination therapies able to overcome chemoresistance and increase 5-FU therapeutic efficiency is one of the major challenges in cancer research.

Some RPs are associated with acquired MDR.^8^ We have previously demonstrated that uL3 downregulation positively correlates with MDR in p53-deleted CRC cells, and uL3 status is essential for cell response to anticancer drugs such as 5-FU, oxaliplatin (OHP), actinomycin D (Act D), and cisplatin.^16−19^ The increased resistance to 5-FU exhibited upon uL3 silencing is associated with increased cell migration and proliferation, apoptosis inhibition, autophagy enhancement, and alteration of the epithelial–mesenchymal transition (EMT) program.^20−22^

Nevertheless, MDR is often related to the alteration in the expression of transporters of the superfamily of ATP-binding cassette (ABC) proteins, which actively expel the drug out of the cells, thereby reducing intracellular drug concentration to below effective levels.^23^

Natural products, like carotenoids, have shown the ability to enhance cancer cell response to cytotoxic drugs and may provide a safer choice to defeat MDR.^24,25^ Among these compounds, β-carotene, a retinol precursor found in fruits and vegetables, has been shown to have antiproliferative and chemopreventive properties.^26−28^ It has been proposed as a chemosensitizer affecting the expression of ABC transporters.^29,30^

The combination of free drugs in solid tumor treatment does not usually exhibit good synergy due to variations in drug properties that affect their pharmacokinetics.^31^ Codelivery of multiple drugs through engineered biodegradable nanoparticles (NPs) can be helpful for improving the therapeutical outcome.^32^ A bright example in this direction is the liposome-encapsulated combination Vyxeos recently approved by the FDA for treating some types of poor prognosis acute myeloid leukemia.^33^ Through appropriate manipulation of composition and overall properties (shell features, size, delivery rate), NPs can become a powerful weapon for implementing combination therapy to overcome MDR.^34^

In the present study, we aimed to investigate the effect of a novel therapeutic strategy to fight MDR in CRC combining β-carotene with 5-FU using nanoparticles as a drug delivery system in a model of 5-FU-resistant CRC cell line deleted of p53 and stably silenced for uL3.^13,16^

We have also investigated the underlying mechanism by which this treatment is able to overcome chemoresistance, providing a new perspective on CRC treatment.

## 2 Experimental Section

### 2.1 Materials

TruSeq RNA Sample Preparation kit and TruSeq PE Cluster Kit v3 were purchased from Illumina, Inc. (San Diego, CA). High Sensitivity DNA Assay kit was purchased from Agilent Technologies, Inc. (La Jolla, CA). Matrigel, ultralow attachment (ULA) round-bottom 96-well plates, and 60 mm tissue culture plates were purchased from Corning (Corning, NY). Dulbecco’s modified Eagle’s medium (DMEM), fetal bovine serum (FBS), and phosphate-buffered saline (PBS) were obtained from HiMedia (Einhausen, Germany). SensiFAST cDNA Synthesis kit and SensiFAST SYBER No-ROX kit were purchased from Meridian Bioscience, Inc. (Cincinnati, Ohio). 5-FU, β-carotene, Act D, RNase, propidium iodide (PI), polyethyleneimine (PEI, 25 kDa branched), poloxamer 188 (Pluronic F68), sodium acetate, sodium chloride, hexadecyltrimethylammonium bromide sodium hydroxide, copper (II) sulfate, tetrahydrofuran (THF), and polysorbate 80 were purchased from Merck KGaA (Darmstadt, Germany). Poly(lactic-*co*-glycolic) acid (PLGA) (d,l-lactic, 50:50 Resomer RG 502H, inherent viscosity 0.16–0.24 dL/g) was obtained from Boehringer Ingelheim (Ingelheim am Rhein, Germany). Formic acid, acetic acid, ethanol, and acetone were purchased from CARLO ERBA Reagents S.r.l. (Milan, Italy). Hyaluronan (HA, <10 kDa) was a kind gift of Magaldi Life S.r.l. (Salerno, Italy). Dialysis bags (MWCO=3500 Da, Spectra/Por) were acquired by Prodotti Gianni (Milan, Italy). Tali Apoptosis Kit was purchased from Life Technologies (Carlsbad, CA). CellTracker Green CMFDA Dye was purchased from Thermo Fisher Scientific Inc. (Waltham, MA). All buffers and solutions were prepared with ultra-high-quality water. All reagents were of the purest commercial grade.

### 2.2 TCGA Database

We extracted the gene expression data and corresponding clinicopathological data for 594 CRC patients from TCGA’s Pan-Cancer Atlas Studies (The Cancer Genome Atlas, https://www.cbioportal.org/).^35^

### 2.3 Library Preparation and Deep Sequencing

For RNA-seq analysis, libraries were prepared according to the manufacturer’s instructions (TruSeq RNA Sample Preparation kit) starting from 4 μg of total RNA. Quality control of library templates was performed using a High Sensitivity DNA Assay kit on a Bioanalyzer (Agilent Technologies, Inc., La Jolla, CA). The Qubit quantification platform (Qubit 2.0 Fluorometer, Life Technologies, Carlsbad, CA) was used to normalize samples for library preparation. Using multiplexing, up to six samples were combined into a single lane to yield sufficient coverage. The sequencing was carried out in collaboration with the Next Generation Sequencing (NGS) Facility at the Telethon Institute of Genetics and Medicine (TIGEM). Cluster generation was performed on Flow Cell v3 (TruSeq PE Cluster Kit v3) using cBOT. Libraries were sequenced by a paired-end chemistry on a NovaSeq. 6000 platform. Each library was loaded at a concentration of 8 pM, which was previously established as optimal. An average yield of ∼4.5 Mb was obtained per sample. The data have been deposited in NCBIs Gene Expression Omnibus (GEO).^36^ GEO accession number is GSE145807.

### 2.4 Computational Analysis of Deep Sequencing Data

A data analysis was performed using the pipeline already established at the Bioinformatics and Statistics Core Facility at TIGEM.^37^ Briefly, the reads were trimmed to remove adapter sequences, and low-quality ends and reads mapping to contaminating sequences (e.g., ribosomal RNA, phIX control) were filtered out. Reads were aligned and assigned to Human ENSEMBLE transcripts and genes (hg38 reference) by using RSEM version 1.2.25 with standard parameters.^38^ The threshold for statistical significance chosen was false discovery rate (FDR) <0.05.

### 2.5 Cell Cultures and Drug Treatments

HCT 116^p53–/–^ cells (American Type Culture Collection, (ATCC) Manassas, Virginia) and uL3ΔHCT 116^p53–/–^ cells, derived from HCT 116^p53–/–^ cell line and stably silenced for uL3,^13^ were cultured as previously described.^39^ Three-dimensional (3D) cultures were established by seeding cells at optimized densities between 1000 and 1500 cells/well in ULA round-bottom 96-well plates in their respective culture media. The cell culture medium of the 3D cocultures was a mixture of DMEM, supplemented with 2.5% Matrigel. Spheroid growth was measured using (i) spheroid size as an indicator of cell viability.

Drug treatments were performed by incubating different formulations of NPs loaded with 5-FU (25 μM), β-carotene (25 μM), or with a combination of these two molecules for 24 or 48 h for two-dimensional (2D) cultures at day 1 post seeding and for 3D cultures at day 1, 2, 4, or 6 post seeding. The cell culture medium and solvent controls were included and used for calculating drug efficacy relative to 100% control.

### 2.6 Quantitative Reverse Transcription Polymerase Chain Reaction (RT-qPCR)

Total RNA was extracted from HCT 116^p53–/–^ and uL3ΔHCT 116^p53–/–^ cells as previously described.^40^ RNA was first retrotranscribed using SensiFAST cDNA Synthesis kit, and then quantitative PCR was carried out using SensiFAST SYBER No-ROX kit. The primers are indicated in Table 1. The comparative Ct method was used to calculate the relative abundance of the mRNA and compared with that of β-actin expression.^41^

**Table 1:** Oligonucleotide Sequences Used in qPCR Analysis

gene	sequence
ABCB1	forward: 5′-GCTGTCAAGGAAGCCAATGCCT-3′
	reverse: 5′-TGCAATGGCGATCCTCTGCTTC-3′
ABCC9	forward: 5′-CAGGCTGTTGGTAGCTCAAGTGCA-3′
	reverse: 5′-ATCTCCACAGCCATCAGCAGCCAAT-3′
ABCC11	forward: 5′-GAAGTCCTCCTTGGGCATGGC-3′
	reverse: 5′-TTATCTCAGTGAAGAAGTGGCTGT-3′
β-actin	forward: 5′-CCAACCGCGAGAAGATGA-3′
	reverse: 5′-CCAGAGGCGTACAGGGATAG-3′

### 2.7 Analysis of mRNA Stability

HCT 116^p53–/–^ and uL3ΔHCT 116^p53–/–^ cells were treated with Act D (5 μg/mL) for 0, 2, 4, 8, 16, and 24 h. Then, total RNA was isolated and the mRNA levels of ABCB1, ABCC9, and ABCC11 were determined by RT-qPCR using specific primers (Table 1). The relative amount of ABCB1, ABCC9, and ABCC11 mRNAs without Act D treatment was set to 100%, and the percentage of these mRNAs treated with Act D was calculated accordingly.

### 2.8 NP Preparation and Characterization

NPs were prepared by solvent diffusion of an organic phase (2 mL) in an aqueous phase (4 mL of water with Pluronic F68 0.1%). The organic phase was prepared by dissolving 10 mg of PLGA 502H and 1 mg of β-carotene in acetone (1.8 mL) and adding 200 μL of a 5-FU stock in ethanol (2.5 mg/mL). After solvent removal under reduced pressure and room temperature, the sample was split into four Eppendorf tubes, centrifuged (5000*g* for 20 min), and redispersed in water (1 mL). Thereafter, 125 μL of a PEI solution (2.5 mg/mL) was added; the samples were washed by centrifugation (2800*g* for 20 min) and redispersed in 1 mL of water. The final NPs were obtained by adding 100 μL of HA in water (1 mg/mL). The interval between each addition was kept constant at 15 min. The hydrodynamic diameter (DH), polydispersity index, and zeta potential (ξ) of NPs were determined on a Zetasizer Nano ZS (Malvern Instruments Ltd.). Results are reported as the mean of three separate measurements on three different batches ± standard deviation (*n* = 9). The yield of the NP production process was evaluated on an aliquot of NP dispersion by weighing the solid residue after freeze-drying. Results are expressed as the ratio of the actual NP weight to the theoretical polymer or polymer + drug weight × 100 ± standard deviation (*n* = 3).

### 2.9 PEI and HA Amount in NPs

PEI was quantified by a colorimetric method developed previously.^42^ To evaluate the amount of PEI in NPs, 0.5 mg of freeze-dried NPs was treated with 1 mL of 1 M NaOH and stirred overnight. The sample (0.5 mL) was diluted with 0.5 mL of 1 M acetic acid. The resulting solution (0.5 mL) was added to 1 mL of 0.1 M acetate buffer at pH 5.4 and complexed with 0.25 mL of a copper(II) sulfate water solution (0.1% w/v). The absorbance value of each solution was recorded at 281 nm (UV-1800, Shimadzu, Japan). A calibration curve was constructed in the same condition in the PEI concentration range of 15–400 μg/mL. The extent of HA adsorption onto NPs was performed according to a previously developed method.^43^ Briefly, 0.5 mg of NPs was centrifuged at 13,000*g* for 15 min; the supernatant was withdrawn and freeze-dried. The solid residue was then dissolved in 1 mL of 0.2 M acetate buffer (0.2 M sodium acetate and 0.15 M sodium chloride) at pH 6. Thereafter, 2 mL of cetyltrimethylammonium bromide reagent (2 g of sodium hydroxide, 1 g of hexadecyltrimethylammonium bromide in 100 mL water) was added and the sample was analyzed at 350 nm. A calibration curve was constructed in the HA concentration range of 10–200 μg/mL.

### 2.10 5-FU and β-Carotene Actual Loading in NPs

5-FU loading inside NPs was assessed by placing 0.5 mg of freeze-dried NPs in 500 μL of DCM and 500 μL of water. Thereafter, the sample was mixed vigorously and centrifuged at 2000*g* for 5 min. The amount of 5-FU in the water phase was analyzed by HPLC as previously described^16^ on a Shimadzu apparatus equipped with an LC-10ADvp pump, a SIL-10ADvp autoinjector, an SPD-10Avp UV–vis detector, and a C-R6 integrator. The analysis was performed on a Synergy Hydro, C18 column (25 × mm). The mobile phase was a 100% (v/v) mixture of water with formic acid (99:1) pumped at a flow rate of 1 mL/min. The UV detector was set at 285 nm. A calibration curve of 5-FU in water was constructed in the concentration range of 1–100 μg/mL.

β-carotene loading inside NPs was evaluated by placing 0.5 mg of freeze-dried NPs in 1 mL of THF. Then, the samples were centrifugated at 13,000*g* for 20 min and the supernatants were analyzed on a UV-1800 spectrophotometer (Shimadzu Corporation, Tokyo, Japan) at 454 nm. A calibration curve of β-carotene in THF was constructed in the concentration range of 0.4–20 μg/mL.

### 2.11 Release Studies of 5-FU and β-Carotene from NPs

In vitro release of 5-FU from NPs was assessed in 10 mM phosphate buffer containing NaCl (137 mM) and KCl (2.7 mM) at pH 7.4 (PBS) by a dialysis method. A known amount of NPs (1.25 mg) was dispersed in 0.5 mL of PBS and placed in a dialysis bag (MWCO = 3500 Da, Spectra/Por). The sample was plunged in 5 mL of PBS (sink condition) and kept at 37 °C. In vitro release of β-carotene was evaluated as described above in 5 mL of PBS containing 10% v/v of polysorbate 80 to ensure sink conditions and avoid β-carotene aggregation. In both cases, at selected time intervals, 1 mL of release medium was withdrawn and replaced with an equal volume of fresh medium. 5-FU or β-carotene quantitative analysis was performed as described above. Results are expressed as release % over time ± standard deviation of three experiments.

### 2.12 Stability of NPs in Fetal Bovine Serum

Stability of NPs in the presence of FBS was assessed by dynamic light scattering (DLS) measurements and turbidimetry analyses. Two hundred microliters of NPs (0.5 mg) were mixed with 800 μL of FBS (10% in water). At different time points (0, 24 h, and 72 h), size, ζ, and scattering (absorbance at 500 nm) of NPs in FBS were monitored. Control samples of FBS and NPs in water were run as control.

### 2.13 Cell Cycle Analysis

uL3ΔHCT 116^p53–/–^ cells were seeded into 60 mm tissue culture plates at a confluency of about 50–60%. Then, cells were starved overnight and treated with NPs loaded with 5-FU (25 μM), β-carotene (25 μM), or with a combination of these two molecules for 24 h. After treatment, the cells (2 × 10^6^) were harvested and centrifuged at 400*g* for 5 min, washed once with cold PBS, and stained in a PI solution as previously reported.^44^ Cell cycle distribution was analyzed using a BD Accuri C6 Plus flow cytometer (BD Biosciences, San Jose, CA).

### 2.14 Cell Death Assay

HCT 116^p53–/–^ and uL3ΔHCT 116^p53–/–^ cells (5 × 10^5^) were seeded into 60 mm tissue culture plates, starved overnight, and treated with NPs loaded with 5-FU (25 μM), β-carotene (25 μM), or with a combination of these two molecules for 48 h. The cells were washed with PBS, harvested by trypsinization, and washed twice with PBS. The cells were then stained with PI and Annexin V Alexa Fluor 488 using Tali Apoptosis Kit according to the manufacturer’s instructions. Briefly, cells were resuspended with 1× binding buffer at a density of 1 × 10^6^ cells/mL. Then, Annexin V Alexa Fluor 488 (5 μL) was added to cell suspension (100 μL) before further incubation for 20 min at RT in the dark. After centrifugation, cells were resuspended with 1× binding buffer (100 μL), stained with PI (1 μL), and analyzed by a BD Accuri C6 Plus flow cytometer. The percentage of Annexin V+/PI– (early apoptosis), Annexin V+/PI+ (late apoptosis), and Annexin V–/PI+ (necrosis) cells was analyzed based on the manufacturer’s instruction. The data are represented as the rate of total apoptotic cells with both early and late apoptotic rates indicated.

### 2.15 3D Spheroid Formation and Growth Analysis

Cells were plated in ULA round-bottom 96-well plates as previously described.^45^ CellTracker Green CMFDA Dye was used to obtain fluorescent-labeled spheroids. Cells were labeled prior to spheroid formation using the manufacturer’s instructions. The spheroid size was measured with the Operetta high-content analysis system (PerkinElmer, U.K.) with the corresponding Image software version 3. A Z-stack of each spheroid was obtained in brightfield and in fluorescence with 5× and 10× objectives, and Z-projection was performed using focus stacking settings. Spheroid size and circularity were calculated using the cellular analysis feature of the software using dark objects on a bright background, do not split touching objects, and a threshold of 15.000 RFU (relative fluorescence unit).

### 2.16 Statistical Analysis

Statistical comparisons were made as previously shown.^46^ Briefly, statistical analyses were performed by GraphPad Prism 6 (La Jolla, CA). Results are expressed as mean ± SD unless indicated otherwise. Groups were compared with either a two-tailed Student’s t-test (for analysis of two groups) or using one-way analysis of variance to compare multiple groups. Significance was accepted when *p* was less than 0.05. The statistical differences of gene expression levels were analyzed by the Mann–Whitney Wilcoxon test or Student’s t-test according to the distribution of the variables. The results were presented as the mean ± standard deviation of samples. We used Pearson’s *R* correlation coefficient to assess relationships between the mRNA expression levels of uL3 and other genes. *p* < 0.05 was considered statistically significant.

## 3 Results

### 3.1 Correlation between uL3 Expression and *ABC* Genes Is Clinically Relevant in CRC Patients

**Figure 1:**
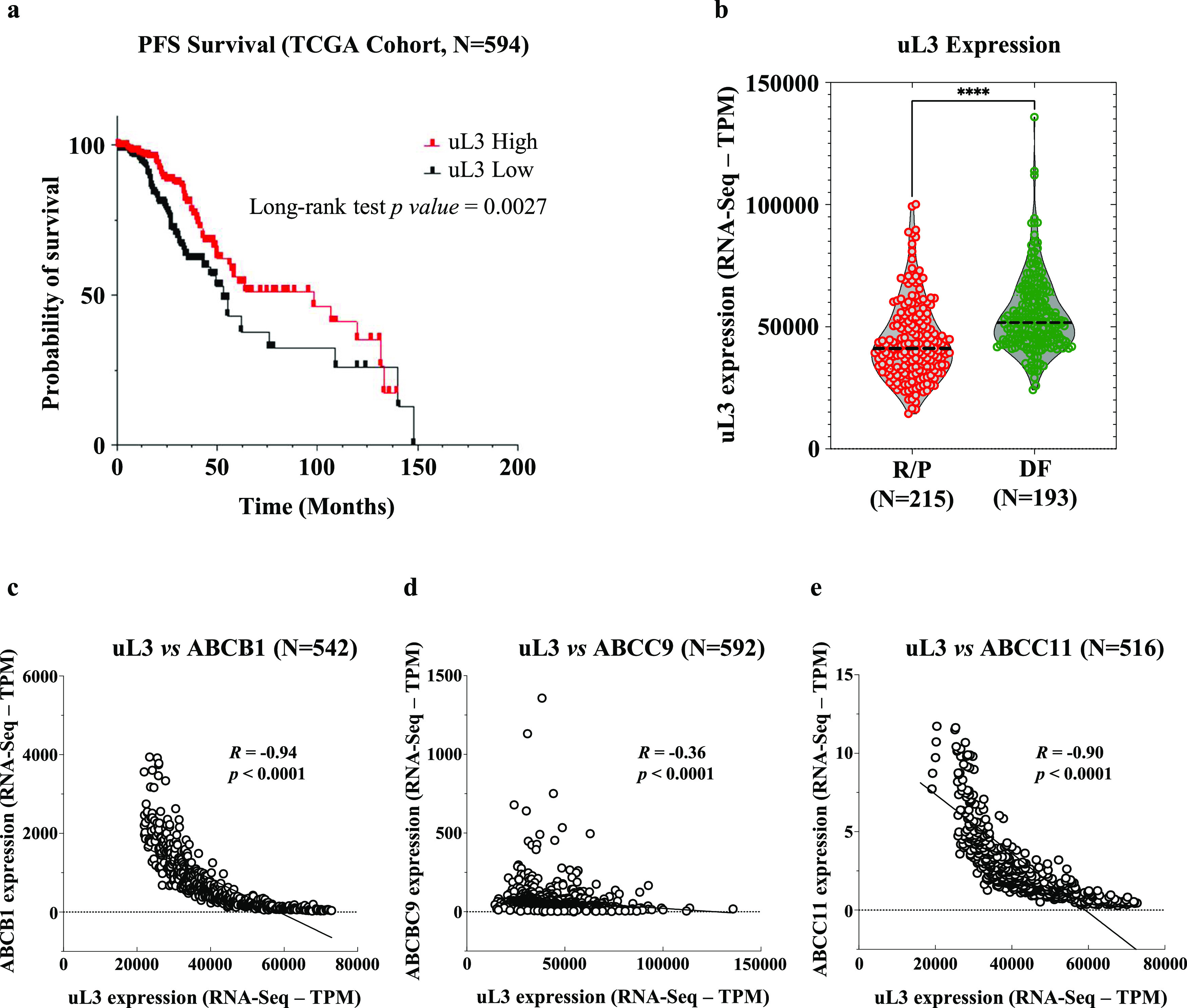
Correlation between uL3 expression and CRC patient’s outcome. (a) RNA-Seq data from the TCGA database were used to stratify the patients into uL3 high and uL3 low levels (median split). Progression-free survival-based (PFS) analysis was performed. (b) The uL3 transcriptional state in recurred or progressed patients (R/P) and disease-free patients (DF). (c–e) Correlation analysis between uL3 expression and levels of specific *ABC* genes.

To evaluate if uL3 expression may influence the CRC patient’s outcome, we analyzed a cohort of 594 colorectal patients (TCGA data sets, https://www.cbioportal.org/)^35^ and divided them into uL3 high- and uL3 low-expression groups (median split). Progression-free survival-based (PFS) analysis revealed that patients in the uL3 high-expressing group had significantly improved PFS, with 50% of this subgroup extending beyond 98 months (Figure 1a). On the contrary, PFS was significantly shorter in the uL3 low group (median 52.9 months vs 98.2 months; *p* = 0.0027 by long-rank test; Figure 1a), indicating that the low expression of uL3 is associated with a poor outcome. By using the best RECIST criterium response, patients were divided into resistant (R/P, recurred or progressed; *n* = 215) and sensitive (DF, disease-free; *n* = 193) to chemotherapeutic. In the TCGA cohort, the uL3 low transcriptional state was found to be associated with poor response to treatments; in fact, the levels of uL3 are significantly decreased in R/P patients (Figure 1b), with respect to DF patients (DF) showing higher expression of uL3 (Figure 1b; *p* < 0.0001). These results reinforced our previous data identifying uL3 as an important player in response to chemotherapeutic drugs^12,17,18^ and suggested a possible application of uL3 as a predictive biomarker of treatment response in CRC.

Furthermore, and consistent with our previous findings,^16^ uL3 levels were found to be inversely correlated with the expression of several *ABC* genes. In particular, we found a strong negative correlation between the mRNA levels of uL3 and ABCB1 (*R* = −0.94, *p* < 0.0001), ABCC9 (*R* = −0.36, *p* < 0.0001), and ABCC11 (*R* = −0.90, *p* < 0.0001) (Figure 1c–e). Importantly, these genes are involved in acquiring MDR to cancer chemotherapeutics, including 5-FU.^23^

These findings, obtained by analyzing the transcriptomes of a large cohort of CRCs, suggest that uL3 may be involved in cancer resistance via ABC gene regulation.

### 3.2 uL3 Regulates the Expression of Specific *ABC* Genes

**Figure 2:**
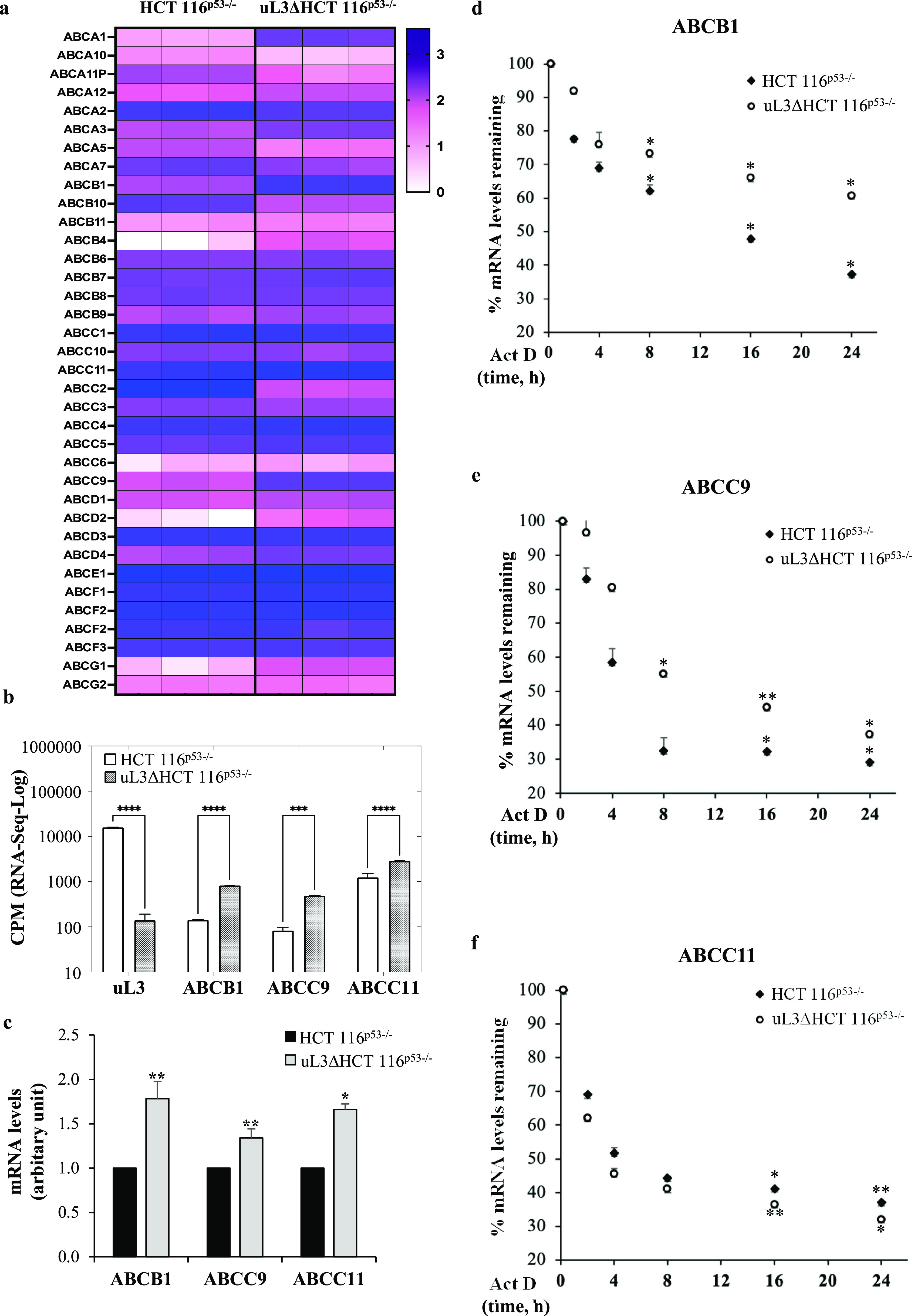
uL3 status affects the expression of specific *ABC transporter* genes in CRC cells. (a) Heat map and cluster analysis of gene expression profiles for 36 ABC transporters in HCT 116^p53–/–^ and uL3ΔHCT 116^p53–/–^ cell lines. Each row represents an *ABC transporter* gene, and each column represents a sample. Genes and samples are arranged according to their similarity in expression levels determined by RNA-Seq. The expression levels of each gene were standardized by subtraction of the mean log 2 (expression level) for the six samples from each value and is colored white, pink, or blue to represent a low, moderate, or high level, respectively. (b) Expression analysis of uL3, ABCB1, ABCC9, and ABCC11 in HCT 116^p53–/–^ and uL3ΔHCT 116^p53–/–^ cells using data retrieved from RNA-Seq. Data were expressed in log-CPM (count per million); ***p < 0.001; ****p < 0.0001. c) RT-qPCR of total RNA extracted from HCT 116^p53–/–^ and uL3ΔHCT 116^p53–/–^ cells with specific primers for ABCB1, ABCC9, ABCC11 and β-actin (Table 1). Error bars represent the standard deviation. *p < 0.05; **p < 0.01 vs HCT 116^p53–/–^cells set at 1.( d-f) HCT 116^p53–/–^ and uL3ΔHCT 116^p53–/–^ cells were treated with Act D (5 μg/mL) and, at indicated time points total RNA was isolated and the mRNA levels of indicated genes were determined by RT-qPCR (Table 1 , (Table 1). Error bars represent the standard deviation. *p < 0.05; **p < 0.01 vs control cells.

To decipher the molecular mechanisms driven by uL3 in drug response, we investigated the whole transcriptome of the CRC cell line silenced for uL3 (uL3ΔHCT 116^p53–/–^), previously characterized for drug resistance,^22^ and the parental cell line (HCT 116^p53–/–^). The differential expression analysis of RNA-Seq data identified *ABC* genes as the most deregulated gene set. The expression profiles of 36 *ABC* genes, in particular, were found to be related to uL3 expression levels (Figure 2a). Among the most deregulated genes, we found *ABCB1*, *ABCC9*, and *ABCC11* (>7-fold; Figure 2b). Their expression was found to be significantly higher in uL3ΔHCT 116^p53–/–^ cells, indicating that uL3 plays an important role in regulating their expression. The expression profile of ABCB1, ABCC9, and ABCC11 was confirmed by RT-qPCR. As shown in Figure 2c, we found that the expression levels of tested genes were strongly increased in uL3ΔHCT 116^p53–/–^ compared to that of parental cells. These results were also confirmed by Western blotting analysis (Figure S1 of the Supporting Information). Overall, these data were consistent with the transcriptomic analysis conducted by RNA-Seq.

To better understand the mechanism by which uL3 regulated the expression levels of selected *ABC* genes, we investigated whether uL3 status could influence the ABCB1, ABCC9, and ABCC11 mRNA stabilities. To this aim, cells were treated with Act D to inhibit nascent RNA synthesis for 2, 4, 8, 16, and 24 h, and mRNA levels were analyzed by RT-qPCR with specific primers for the investigated ABC transporters (Table 1). The results showed that in HCT 116^p53–/–^ the amounts of ABCB1 and ABCC9 mRNAs were lower than in uL3ΔHCT 116^p53–/–^ at all time points (Figure 2d,e), whereas the amount of ABCC11 mRNA did not change significantly in uL3-silenced CRC cells compared to that of parental cells, demonstrating that the uL3 status did not affect ABCC11 mRNA stability (Figure 2f).

These data indicate that the upregulation of ABCB1 and ABCC9 mRNA levels observed in uL3ΔHCT 116^p53–/–^ cells could be partly due to an increase in mRNA stability. Overall, we can conclude that uL3 functions as a transcriptional (ABCC11) and post-transcriptional (ABCB1 and ABCC9) regulator of *ABC* genes, influencing their expression levels and, as a result, cellular drug response.

### 3.3 Delivery of 5-FU and β-Carotene to CRC Cells with Targeted Nanocarriers

Aiming to identify novel therapeutic strategies to overcome drug resistance and in the light of our previous results, we decided to verify the efficiency of a combinational therapy of 5-FU plus β-carotene in our model of resistant CRC, uL3ΔHCT 116^p53–/–^ cells. For the codelivery of multiple drugs, NPs have emerged as a promising class of carriers.^32^ Packing 5-FU and β-carotene (β-car) into a single nanocarrier enables drug release in cancer cells at precisely tuned ratios and rates.

**Figure 3:**
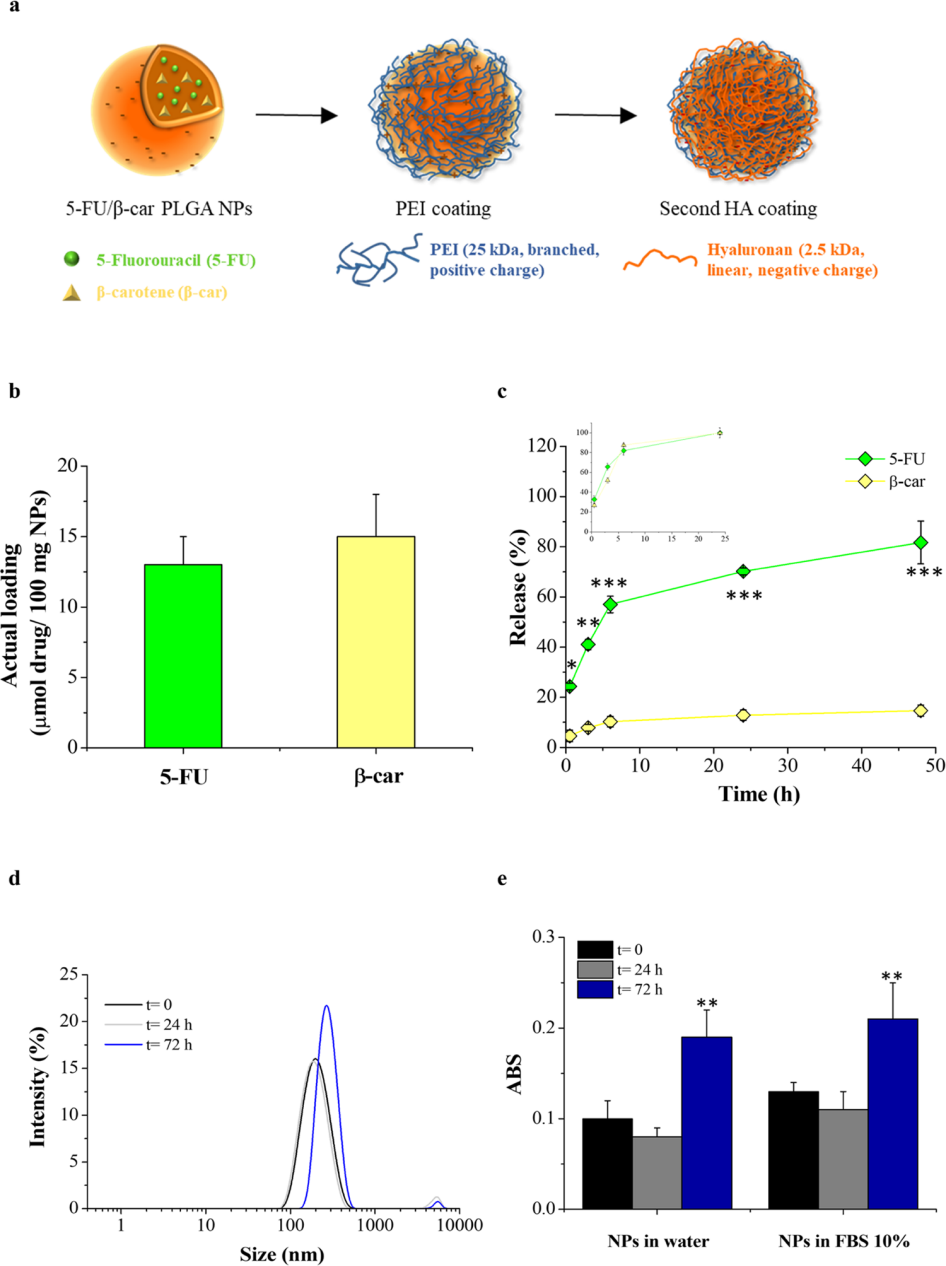
NP design and properties. (a) Schematic illustration of NP structure and preparation through the layering procedure. (b) Entrapment of 5-FU and β-carotene inside NPs. (c) Release kinetics of β-carotene and 5-FU from NPs in PBS pH 7.4 with or without polysorbate 80 v/v, respectively, and 37 °C. In the inset, the release of the free drugs is reported as controls. *p < 0.05; **p < 0.01; ***p < 0.001 vs β-carotene release. (d) Size distribution curves of NPs (0.5 mg/mL) in FBS at 10% at different time points. (e) Scattering of NPs in water and in FBS at 10% and 37 °C at different time points. Results are the mean of three measurements obtained on three different NP batches ± standard deviation. **p < 0.01 vs NPs at time 0.

We prepared double-coated NPs based on a PLGA lipophilic core entrapping both 5-FU and β-carotene and covered by an external shell of negatively charged HA through a cationic bridging layer of PEI (Figure 3a). Our previous results demonstrated that the CD44 receptor mediates endocytosis of these HA-coated NPs in HCT 116^p53–/–^ cells.^16^ Due to their versatility, double-coated NPs have been tested in other CD44-positive cancer models for the targeted delivery of chemotherapeutic combinations.^42,43^

We prepared a panel of NPs loaded with both drugs (5-FU/β-car/NPs) and, as controls, both NPs loaded with a single drug at the same concentration (5-FU/NPs and β-car/NPs) and unloaded NPs. Their properties are reported in Table 2. All of the formulations displayed a size around 200 nm, a polydispersity index <0.2, and a negative zeta potential due to the presence of the external layer of HA. The yield of the production process was around 50%, which is satisfactory for a lab scale due to the loss of some NPs during the purification steps. The higher entrapment efficiency of β-carotene compared to that of 5-FU was in line with its higher lipophilicity and solubility in the PLGA matrix. The actual loading of 5-FU and β-carotene was 13 and 15 μmol for 100 mg of NPs, respectively, as reported in Figure 3b. The release profile of the drugs from NPs was evaluated by dialysis using PBS at pH 7.4 as both the internal and external media. In particular, to set sink conditions, the release of β-carotene was performed in PBS enriched with polysorbate 80. A slow and sustained release of both components from NPs was found (Figure 3c). In particular, 5-FU release was faster than that of β-carotene and completed after 2 days of incubation. On the contrary, at the same time, only 20% of the loaded β-carotene was released, thus confirming its higher affinity with the PLGA core (Figure 3c).

**Table 2:** Colloidal Properties of NPs

batch	size (nm ± SD)	ζ (mV ± SD)	P.I.	yield (%)
Unloaded NPs	196 ± 8	–15 ± 2	0.2	54
5-FU/NPs	231 ± 7	–12 ± 3	0.2	50
β-car/NPs	226 ± 8	–13 ± 2	0.2	48
5-FU/β-car/NPs	205 ± 9	–12 ± 2	0.2	56

To mimic NP behavior in cell culture experiments and evaluate the contribution of proteins, we tested the stability of NPs in the presence of FBS at 10%, i.e., the concentration employed in cell culture media. We measured the size of NPs (Figure 3d) and the turbidity of the dispersion (Figure 3e) after 24 and 72h of incubation. As clearly evidenced by the figures, size growth in the presence of proteins did not give any macroscopic NP aggregation over time. NPs showed excellent stability up to 24 h of incubation, whereas a slight interaction with FBS was found at 72 h, probably due to the repulsion forces of the HA.

Next, we investigated the involvement of the CD44 receptor in the uptake of HA-coated NPs in uL3ΔHCT 116^p53–/–^ cells. To this purpose, we first evaluated the expression levels of the CD44 receptor in these cells by Western blotting analysis. Figure S2 of the Supporting Information shows that uL3ΔHCT 116^p53–/–^ cells express the CD44 receptor when compared to a pancreatic cell line used as a positive control.^47^ For competitive binding experiments, cells were preincubated with excess free HA to saturate CD44 receptors and were subsequently treated with rhodamine B-labeled NPs. The amount of internalized NPs was analyzed by fluorimetry. Our results demonstrated the reduction of NP uptake caused by free HA (Figure S2b of the Supporting Information).

### 3.4 β-Carotene Plus 5-FU Affect Cell Cycle Progression in CRC Cells Silenced for uL3

**Figure 4:**
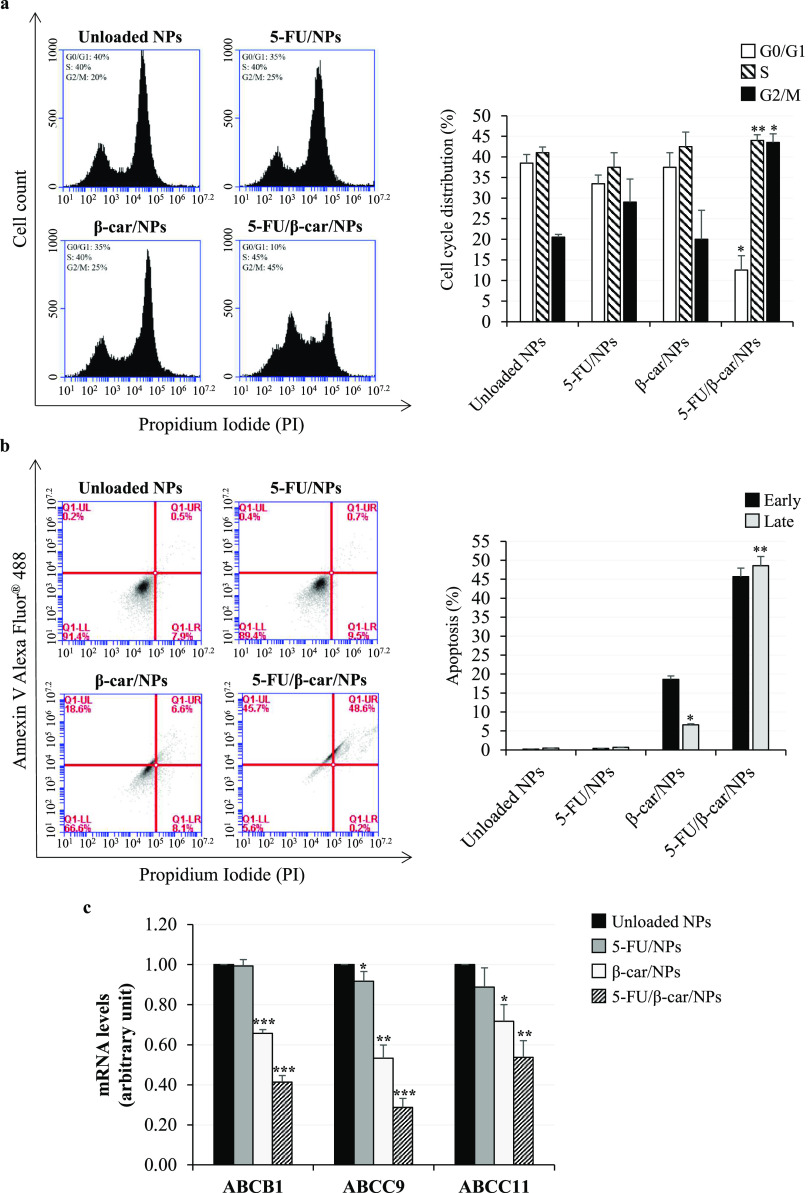
Effect of 5-FU and β-carotene treatment on cell cycle, apoptosis, and expression of specific *ABC transporter* genes in uL3ΔHCT 116^p53–/–^ cells. (a) Representative FACS histograms of PI-stained cells treated with unloaded NPs, 5-FU/NPs, β-car/NPs, or 5-FU/β-car/NPs for 24 h. The bar diagram shows the percentage of cells in each phase of the cell cycle. For each sample, at least 2 × 10^4^ events were analyzed. (b) Representative flow cytometry dot blots with double Annexin V Alexa Fluor 488/PI staining for cells treated with unloaded NPs, 5-FU/NPs, β-car/NPs, or 5-FU/β-car/NPs for 48 h. The bar diagram shows the percentage of early (Annexin V+/PI-) and late (Annexin V+/PI+) apoptotic cells. For each sample, at least 2 × 10^4^ events were analyzed. (c) Cells were treated with unloaded NPs, 5-FU/NPs, β-car/NPs, or 5-FU/β-car/NPs for 24 h. Total RNA was isolated, and the mRNA levels of ABCB1, ABCC9, ABCC11, and β-actin (Table 1) were determined by RT-qPCR. Bars represent the mean of triplicate experiments; error bars represent the standard deviation. *p < 0.05, **p < 0.01, and ***p < 0.001 vs unloaded NP-treated cells set at 1.

Previous studies showed that 5-FU exerts an anticancer effect by affecting the cell cycle.^48^ Therefore, the cell cycle distribution of our panel of NPs was investigated. uL3ΔHCT 116^p53–/–^ cells were treated with NPs loaded with 5-FU (5-FU/NPs), β-carotene (β-car/NPs), or with a combination of these two molecules (5-FU/β-car/NPs). After 24 h, the cell cycle was analyzed by flow cytometry. As shown in Figure 4a, the cell cycle profile revealed that 5-FU/NPs and β-car/NPs did not affect cell cycle distribution in uL3ΔHCT 116^p53–/–^ cells (Figure 4a). Surprisingly, uL3ΔHCT 116^p53–/–^ cells, previously shown to be resistant to 5-FU,^13^ became sensitive when treated with 5-FU/β-car/NPs and displayed a cell cycle arrest in the G2/M phase (Figure 4a). These findings suggest that combining β-carotene and 5-FU may restore 5-FU sensitivity in resistant CRC cells.

### 3.5 β-Carotene Plus 5-FU Reverse Chemoresistance in CRC Cells Silenced for uL3

To better investigate the biological behavior of our NPs, we assessed their cytotoxicity on uL3ΔHCT 116^p53–/–^ using the CCK-8 assay. To this aim, cells were incubated for 48 h with NPs loaded with different concentrations of 5-FU (from 1.6 to 25.0 μM), or β-carotene (from 1.6 to 25.0 μM) or a combination of 5-FU and β-carotene at the same concentrations (Figure S3 of the Supporting Information). The results of these assays show that cells retained about 90% viability when treated with 5-FU/NPs compared to the control (untreated cells set to 100%), confirming the 5-FU resistance of this cell line. Treatment with β-car/NPs resulted, even at higher β-carotene concentrations (25.0 μM), in 70% viability indicating that this molecule has a slight cytotoxic effect. Interestingly, the combined treatment (5-FU/β-car/NPs) significantly reduced cell viability in a dose-dependent manner. Specifically, we observed that the combined treatment reduced cell viability by 40% at 3.1 μM and of 90% at 25 μM (Figure S3 of the Supporting Information).

Starting from these results,^64^ we wished to investigate the influence of the combined strategy on apoptosis. To this purpose, we carried out an Annexin V/PI dual staining of uL3ΔHCT 116^p53–/–^ and HCT 116^p53–/–^ cells treated with the NPs for 48 h (Figures 4b and S4 of the Supporting Information). As shown in Figure 4b, the treatment of uL3ΔHCT 116^p53–/–^ cells with 5-FU/NPs failed to induce apoptosis, whereas a little proportion of late apoptotic cells was observed with β-car/NPs (7% compared to control cells). Interestingly, the 5-FU/β-car/NPs caused a significant increase in the percentage of late apoptotic cells (49% compared to control cells).

In addition, the treatment of HCT 116^p53–/–^ cells with 5-FU/NPs and β-car/NPs significantly increased the percentage of late apoptotic cells by approximately 16 and 15%, respectively, compared to control cells (Figure S4 of the Supporting Information). Interestingly, the combination of 5-FU and β-carotene (5-FU/β-car/NPs) induced a strong increase in the percentage of late apoptotic cells by approximately 30%, suggesting an additive effect of 5-FU and β-carotene in CRC cells.

Overall, these results show that the 5-FU/β-car/NP treatment can resensitize resistant CRC cells to 5-FU treatment and can positively modulate apoptosis in both sensitive and resistant CRC cells.

### 3.6 β-Carotene Plus 5-FU Modulate the Expression of Specific *ABC* Genes in CRC Cells Silenced for uL3

To understand if the efficiency of the combined treatment in sensitizing chemoresistant CRC cells was due to the regulation of the ABC transporter expression, uL3ΔHCT 116^p53–/–^ cells were treated with 5-FU/NPs, β-car/NPs, or 5-FU/β-car/NPs. The total RNA was extracted after 24 h, and expression of ABCB1, ABCC9, and ABCC11 was analyzed by RT-qPCR with specific primers. As shown in Figure 4c, in uL3ΔHCT 116^p53–/–^ cells, the expression levels of all tested genes did not change significantly upon the treatment with 5-FU/NPs. In contrast, 5-FU/β-car/NPs caused a strong downregulation of ABCB1, ABCC9, and ABCC11 expressions. Notably, a decrease in the expression levels of these transporters was observed upon treatment with β-car/NPs, suggesting that β-carotene could contribute to resensitize resistant cells to 5-FU through to the downregulation of ABC transporters (Figure 4c).

### 3.7 5-FU in Combination with β-Carotene Inhibits Spheroid Culture Growth

Three-dimensional (3D) cultures can potentially increase the predictive value of preclinical drug research and bridge the gap toward a clinical outcome of the proposed strategy.^49^

**Figure 5:**
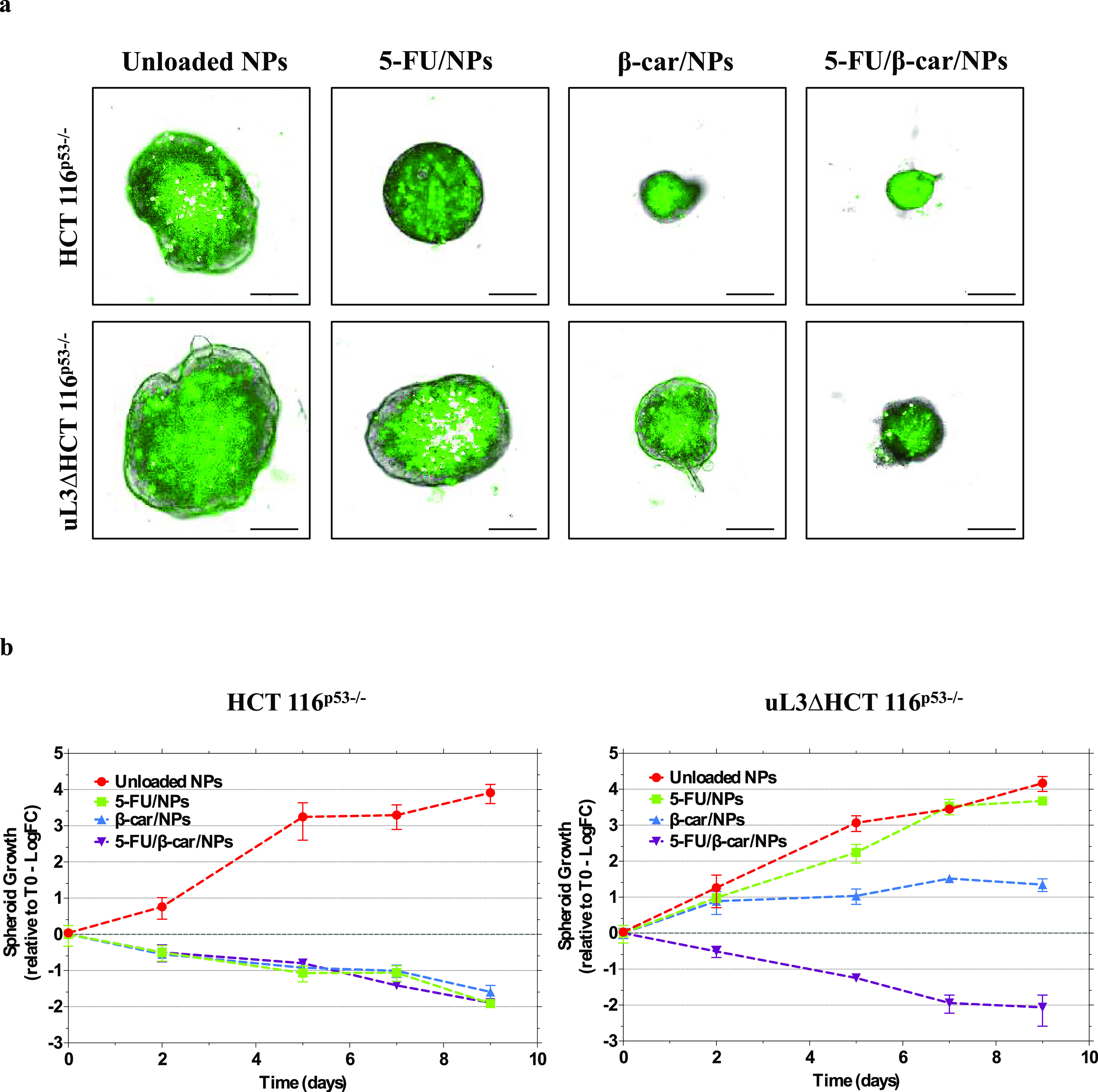
Effect of 5-FU and β-carotene treatment on spheroid growth. (a) Representative phase contrast and overlaid fluorescence images of the CRC spheroids grown at the optimal seeding densities at day 5. Spheroids were established from HCT 116^p53–/–^ and uL3ΔHCT 116^p53–/–^ cells. After 2 days of culture, spheroids were treated with unloaded NPs, 5-FU/NPs, β-car/NPs, and 5-FU/β-car/NPs. Growth medium with treatments was replaced every 2 days. Scale bar represents 100 μm. (b) Volumetric measurements of spheroid growth over 8 days of culture. Data are represented as log fold change relative to day 0 (T0). Error bars represent the standard deviation, *n* = 3–6.

HCT 116^p53–/–^ and uL3ΔHCT 116^p53–/–^ cells were selected for further investigation, and their spheroids were exposed for 9 days to 5-FU/NPs, β-car/NPs, or 5-FU/β-car/NPs. We monitored the effects of each treatment after 24 h when compact 3D spheroids were formed. The analysis was performed by a high-content confocal imaging platform, a technology that combines automated fluorescence microscopy with automated image analysis and allows tracking of cellular morphology and intracellular parameters. The spheroid volume as a readout in long-term cultures revealed significant differences between the treatments over time (Figure 5). As expected, 3D cultures of HCT 116^p53–/–^ cells were significantly more sensitive to all of the NP formulations tested. In contrast, the analysis of the 3D kinetic growth of uL3ΔHCT 116^p53–/–^ cells revealed no significant response to 5-FU/NPs (Figure 5), confirming that this cell line is resistant to 5-FU. The treatment with NPs loaded with 5-FU/β-car/NPs, and to a lower extent with β-car/NPs, showed a significant efficiency in inhibiting spheroid growth.

Taken together, these findings demonstrate that the 5-FU/β-car/NP treatment can overcome the drug resistance induced by uL3 silencing also in a 3D tumor growth model, offering a novel therapeutic approach for treating chemotherapy-resistant CRCs.

## 4 Discussion

CRC is one of the leading causes of cancer-related death worldwide and requires surgical intervention and chemotherapy.^50^ Although the response rate to systemic chemotherapies is high, drug resistance develops frequently, limiting the therapeutic efficacy of anticancer drugs.^51^ High heterogeneity among tumors and the high complexity of the evolution of tumor progression need the identification of novel strategies to improve therapeutic outcomes and overcome drug resistance. In particular, individual differences in mutations of cancer-related genes require personalized therapeutic approaches.^52,53^

Emerging targets in cancer drug resistance include RPs. Among these, we have demonstrated that uL3 exerts extra-ribosomal functions^8^ and its downregulated expression in CRC cells causes chemoresistance by increasing autophagic flux and inhibiting apoptosis.^16,54^

Currently, the standard chemotherapy for CRC includes 5-FU.^6^ One crucial reason for 5-FU treatment failure is the development of acquired MDR.^15^ Drug resistance toward antineoplastic agents is mainly a result of a reduction in the intracellular drug concentration in the cell due to the altered expression of drug transporters.^23^

In this paper, we have investigated the potential predictive role of uL3 in CRC response to chemotherapy and analyzed the efficiency of a combination therapy of 5-FU and β-carotene to overcome MDR in CRC cells. To accomplish this, we first investigated the relationship between the uL3 expression profile, patient outcome, and *ABC* gene expression in a large cohort of human CRC samples (*n* = 594). We found that the reduced uL3 levels were associated with poor response to therapeutic treatment and higher *ABC* gene expression (Figure 1). To recapitulate these findings *in vitro*, we validated the cell model employed for this study. Transcriptome analysis of CRC cells silenced for uL3 (uL3ΔHCT 116^p53–/–^ cells) and resistant to 5-FU revealed an increased expression of three specific ABC transporters, namely, ABCB1, ABCC9, and ABC11 (Figure 2a–c), which ensures higher amounts of the related transporters on the cell membrane. At the molecular level, the downregulation of uL3 is associated with ABCB1 and ABCC9 positive regulations of mRNA stability (Figure 2d,e).

Overall, these findings suggest that uL3 could represent a valuable biomarker for predicting tumor recurrence and patient outcome and that regulating *ABC* gene expression could be convenient for CRC therapy.

Many natural products have shown the ability to regulate the expression of ABC transporters and may sensitize resistant cells to drugs.^55,56^ Among them, β-carotene has been shown to reverse MDR in cancer cells by interfering with the expression of ABC transporters. Therefore, we designed and developed a codelivery system transporting both 5-FU and β-carotene for a combination therapy able to overcome uL3-related drug resistance in CRC therapy and tested its efficacy in 2D and 3D cell models.

In the arena of combination therapy, nanotechnology is considered crucial since it allows the precise delivery of different drugs with different molecular mechanisms at the target site at appropriate ratios.^32^ A further reason for using a nanocarrier is the possibility of finding a suitable vehicle for such a lipophilic drug as β-carotene that can be solubilized only in highly lipophilic solvents. The concept of delivering drug combinations in a single nanocarrier is very appealing to synchronize the pharmacokinetics of drugs with different properties (blood–protein interaction, hydrophilicity, p*K*_a_) and drive them to the same targeted region at a predetermined ratio. This aspect is highly relevant since synergistic or additive effects occur only at specific drug ratios, as demonstrated in previous studies.^57,58^ Instead, the accumulation of a mixture of NPs bearing a different drug cargo is much less predictable and affected by the biological environment the nanoplatform faces. Double-coated PLGA NPs have a core of PLGA, an FDA-approved polymer, widely employed for its safe toxicity profile.^59^ NPs are surface-modified with HA to target the CD44 receptor (Figure S2 of the Supporting Information).

Data presented in this paper indicated that neither 5-FU/NPs nor β-car/NPs had a significant effect on cell cycle or apoptosis in uL3-silenced CRC cells. Notably, the combination of β-carotene and 5-FU (5-FU/β-car/NPs) caused a cell cycle arrest in the G2/M phase and strongly activated apoptosis (Figure 4). These findings indicate that β-carotene acts as a sensitizer and that the combined use of 5-FU and β-carotene, delivered appropriately by NPs, may enable the overcoming of chemoresistance in our model of CRC cells.

Next, to better mimic the in vivo environment, the physiological cell–cell, and cell–matrix interactions that occur in solid tumors, 3D spheroid cultures were generated. Even though spheroids lack vasculature and clonal evolution, 2D- and 3D-cultured cells show different gene expression profiles and molecular fingerprints more closely recapitulating the features of patient tumors.^60^ Several studies have shown that 2D and 3D cultures react differently to anticancer drugs.^61^ The variability of drug susceptibility is usually explained by different “microenvironments” and gene expression profiles, apart from nutrient and oxygen gradients and diffusion capacity of the drug.^62^ It has also been shown that spheroids of some cell lines can exhibit resistance to cytotoxic drugs including 5-FU. It can be assumed that the main factor that contributes to drug resistance is the insufficient penetration and distribution of the drugs in the spheroid cell mass.^63^ Our data demonstrated that the proposed combined treatment is successful also in spheroids of uL3-silenced CRC cells (Figure 5), demonstrating the major ability of NPs to deliver both 5-FU and β-carotene in the spheroid cell mass.

Future studies in an in vivo experimental model will be required to verify the effectiveness of the proposed combination therapy. We will examine the therapeutic effects of the suggested treatment in a xenograft mouse model derived from CRC cell lines that have been silenced for uL3, with a focus on the biodistribution and metabolism of both molecules.

## 5 Conclusions

**Figure 6:**
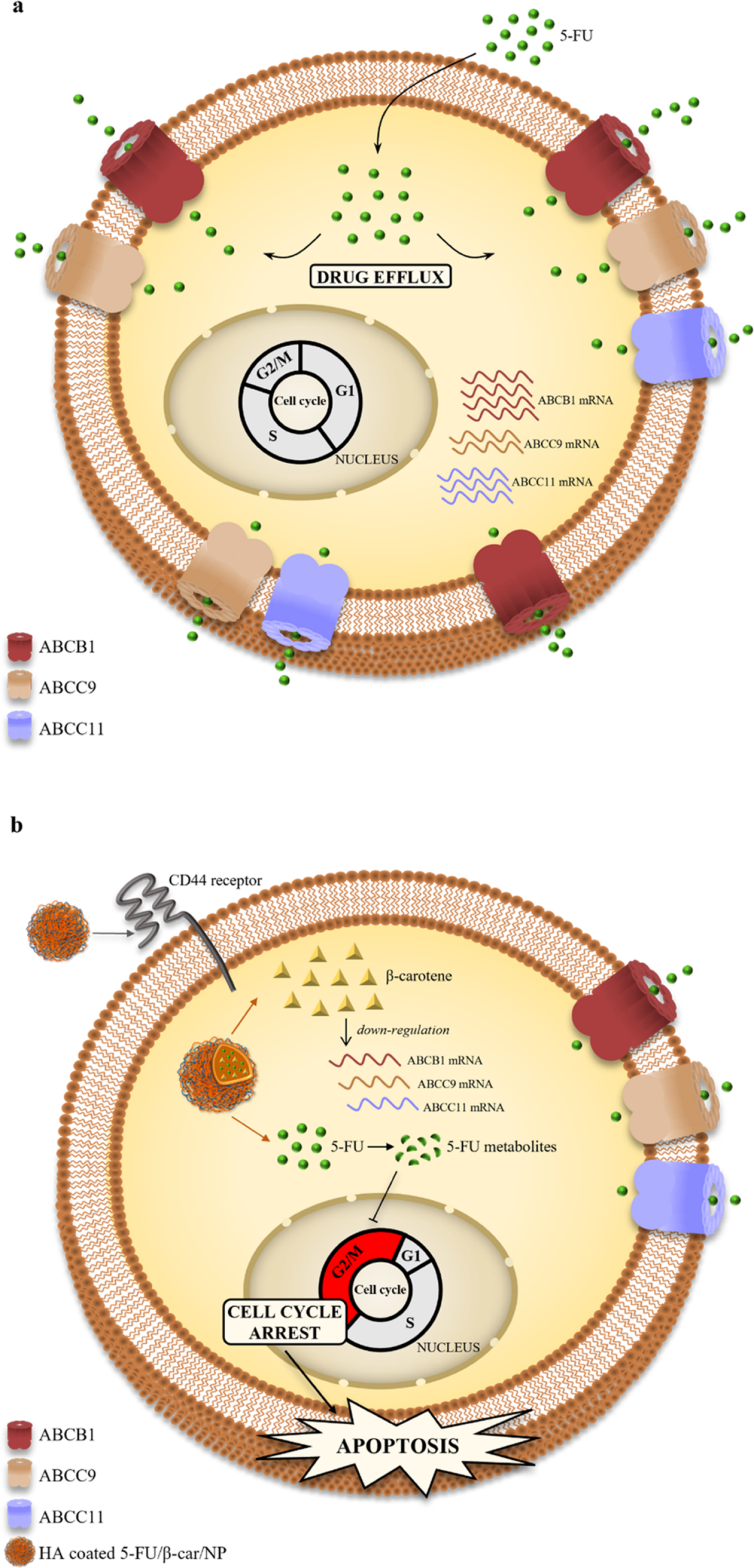
Schematic representation of the proposed model. (a) Overexpression of specific ABC transporters as a mechanism of uL3-mediated 5-FU resistance. 5-FU efflux from resistant uL3ΔHCT 116^p53–/–^ cells is due to the overexpression of ABCB1, ABCC9, and ABCC11 transporters. (b) The combined strategy of 5-FU/β-car/NPs overcomes ABC transporter efflux mechanism of uL3-mediated resistance to 5-FU. HA-coated NPs through CD44 receptor-mediated endocytosis release β-carotene and 5-FU into the cell. β-carotene negatively regulates the expression of ABCB1, ABCC9, and ABCC11 transporters contributing to avoid 5-FU efflux leading to cell cycle arrest in the G2/M phase and apoptosis.

Overall, our data led us to propose a working model of uL3-based drug resistance in which the failure of 5-FU treatment of resistant cells, silenced for uL3, is mainly due to the overexpression of specific ABC transporters that pump the 5-FU outside of cancer cells, making it ineffective (Figure 6a). HA-based NPs can effectively accumulate in CRC cells through CD44-mediated uptake and release 5-FU and β-carotene intracellularly. Thereafter, β-carotene downregulates ABCB1, ABCC9, and ABCC11 mRNA and the corresponding transporter levels, increasing the intracellular amount of 5-FU and causing cell cycle arrest in the G2/M phase and apoptosis (Figure 6b).

In conclusion, this study strongly indicates that double-coated NPs delivering 5-FU and β-carotene to CRC cells represent a novel promising therapeutic strategy to tackle resistant CRCs.

## Data Availability

The data sets generated and/or analyzed during the current study are available in the GEO database (GEO accession number GSE145807).

## Supporting Information Available

Expression analysis of ABCB1, ABCC9, and ABCC11 in HCT 116^p53–/–^ and uL3ΔHCT 116^p53–/–^ cells (Figure S1); expression analysis of CD44 receptor and cellular uptake of rhodamine B-labeled NPs in uL3ΔHCT 116^p53–/–^ cells (Figure S2); cytotoxicity of 5-FU/NPs, β-car/NPs, and 5-FU/β-car/NPs in uL3ΔHCT 116^p53–/–^ cells (Figure S3); effect of 5-FU and β-carotene treatment on apoptosis in HCT 116^p53–/–^ cells (Figure S4); full-length blots of Figure S1 (Figure S5); and full-length blots of Figure S2a (Figure S6) (PDF)
