# Supplementary material for: Combining β-Carotene with 5-FU via Polymeric Nanoparticles as a Novel Therapeutic Strategy to Overcome uL3-Mediated Chemoresistance in p53-Deleted Colorectal Cancer Cells
Source: Mol Pharm. 2023 Mar 28;20(5):2326–40. doi: 10.1021/acs.molpharmaceut.2c00876 (PMC10155186; doi:10.1021/acs.molpharmaceut.2c00876)
Supplement: Supplementary file 1 — mp2c00876_si_001.pdf [file mp2c00876_si_001.pdf]

Combining β-Carotene with 5-FU via Polymeric Nanoparticles as a Novel Therapeutic Strategy to Overcome uL3-Mediated Chemoresistance in p53-Deleted Colorectal Cancer Cells

Pietro Carotenuto,[⊥1,2] Annalisa Pecoraro,[⊥3] Chiara Brignola,[3] Anna Barbato,[1] Brunella Franco,[1,2,4] Giuseppe Longobardi,[3] Claudia Conte,[*3] Fabiana Quaglia,[3] Giulia Russo,[3] and Annapina Russo[*3]

[1] TIGEM, Telethon Institute of Genetics and Medicine, 80078 Naples, Italy

[2] Medical Genetics, Department of Translational Medical Science, University of Naples “Federico II”, 80131, Naples, Italy

[3] Department of Pharmacy, University of Naples “Federico II”, 80131, Naples, Italy

[4] Scuola Superiore Meridionale, School for Advanced Studies, 80138, Naples, Italy

⊥ These authors equally contributed to the paper

* Correspondence to: Annapina Russo and Claudia Conte

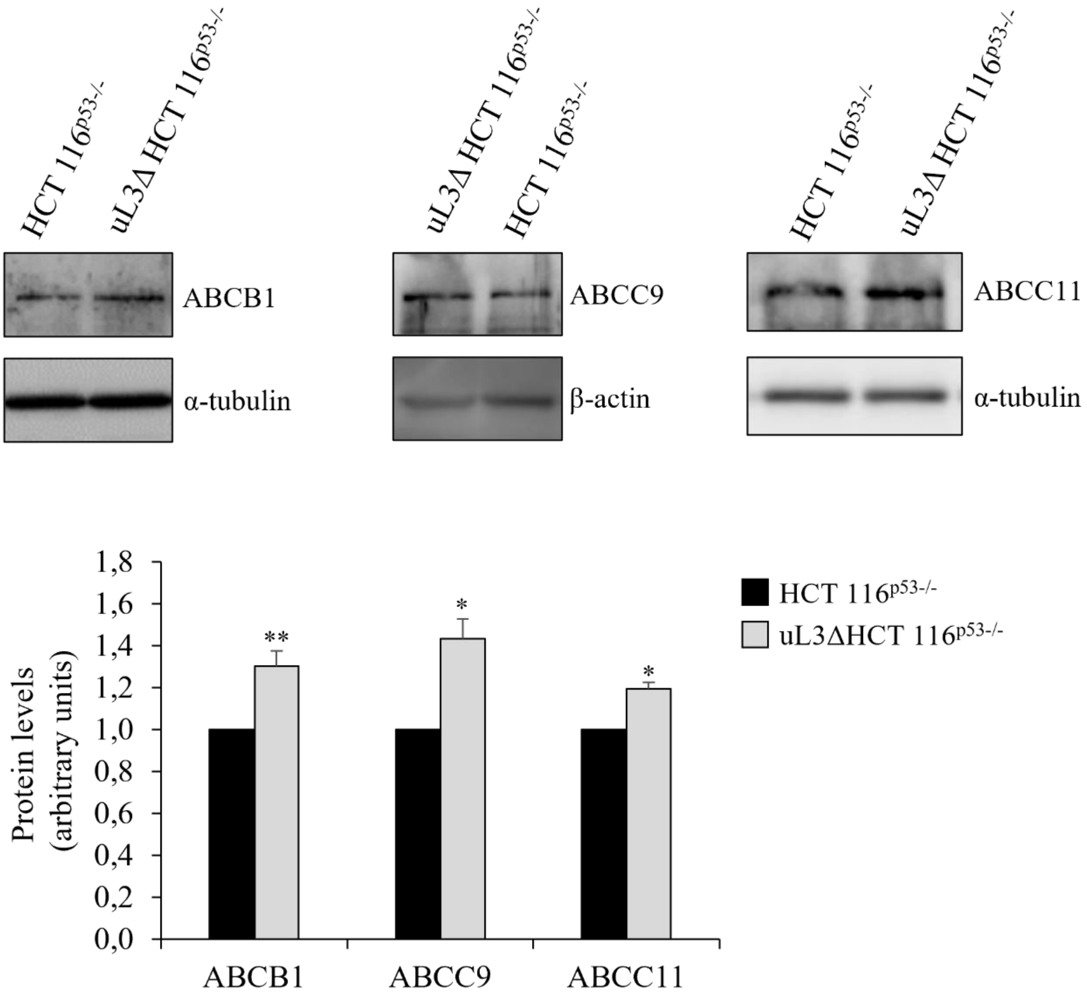

Figure S1. Expression analysis of ABCB1, ABCC9 and ABCC11 in HCT 116$^{p53-/-}$ and uL3ΔHCT 116$^{p53-/-}$ cells. Protein extracts were analyzed by western blotting with the indicated antibodies. *α*-tubulin and *β*-actin were used as loading controls. Full length blots are presented in Figure S5. Quantification of signals is shown. Bars represent the mean of triplicate experiments; error bars represent the standard deviation. *$p < 0.05$; **$p < 0.01$ vs HCT 116$^{p53-/-}$ cells set at 1.

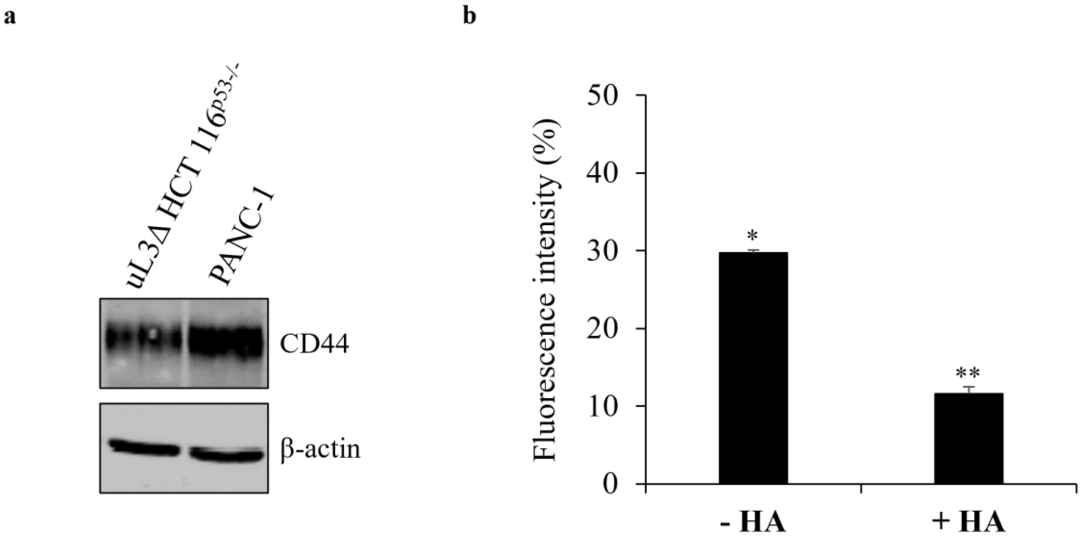

Figure S2. Expression analysis of CD44 receptor and cellular up-take of Rhodamine B-labeled NPs in uL3ΔHCT $116^{p53-/-}$ cells. (a) Protein extracts from uL3ΔHCT $116^{p53-/-}$ and PANC-1 (positive control) cells were analyzed by western blotting with the indicated antibodies. *β*-actin was used as loading controls. Full length blots are presented in Figure S6. (b) Cells were pre-treated or not with free HA for 1 h to block the CD44 receptor. Then, cells were incubated with Rhodamine B-labeled NPs for 2 h. Bars represent the mean of triplicate experiments; error bars represent the standard deviation. **p* < 0.05; ***p* < 0.01 vs control set as 100%.

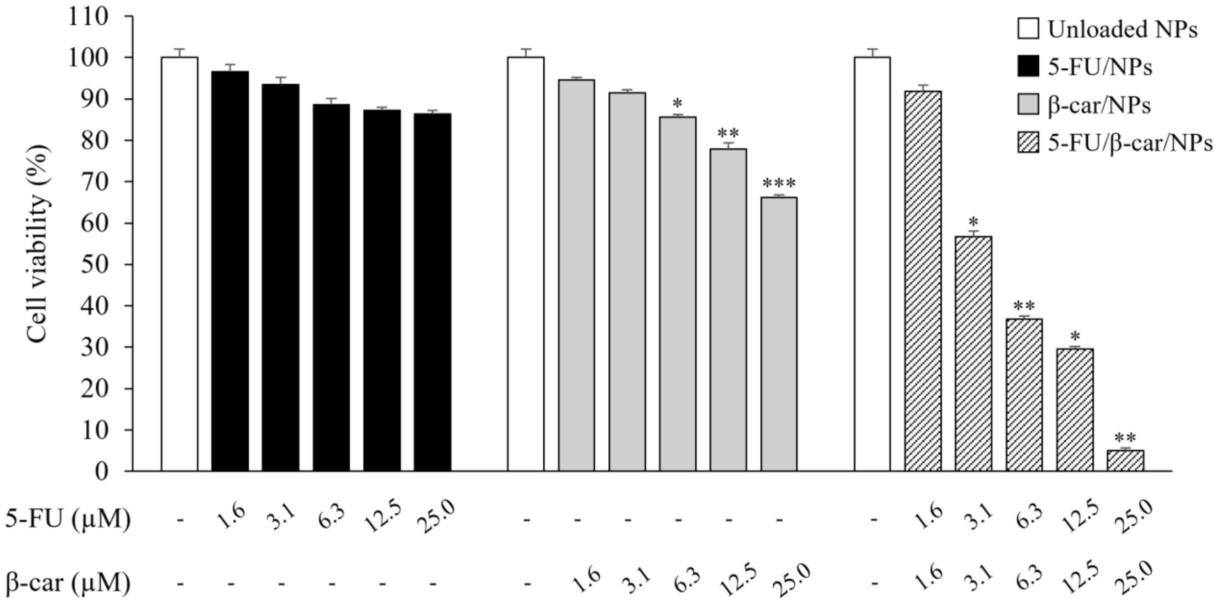

Figure S3. Cytotoxicity of 5-FU/NPs, *β*-car/NPs and 5-FU/*β*-car/NPs in uL3ΔHCT 116$^{p53-/-}$ cells. Cells were treated with unloaded NPs or NPs loaded with a wide range of 5-FU and/or *β*-carotene (from 1,6 to 25,0 μM). After 48 h, cell viability was evaluated by CCK-8 assay. Bars represent the mean of triplicate experiments; error bars represent the standard deviation. *$p < 0.05$; **$p < 0.01$; ***$p < 0.001$ vs unloaded NP-treated cells set as 100%.

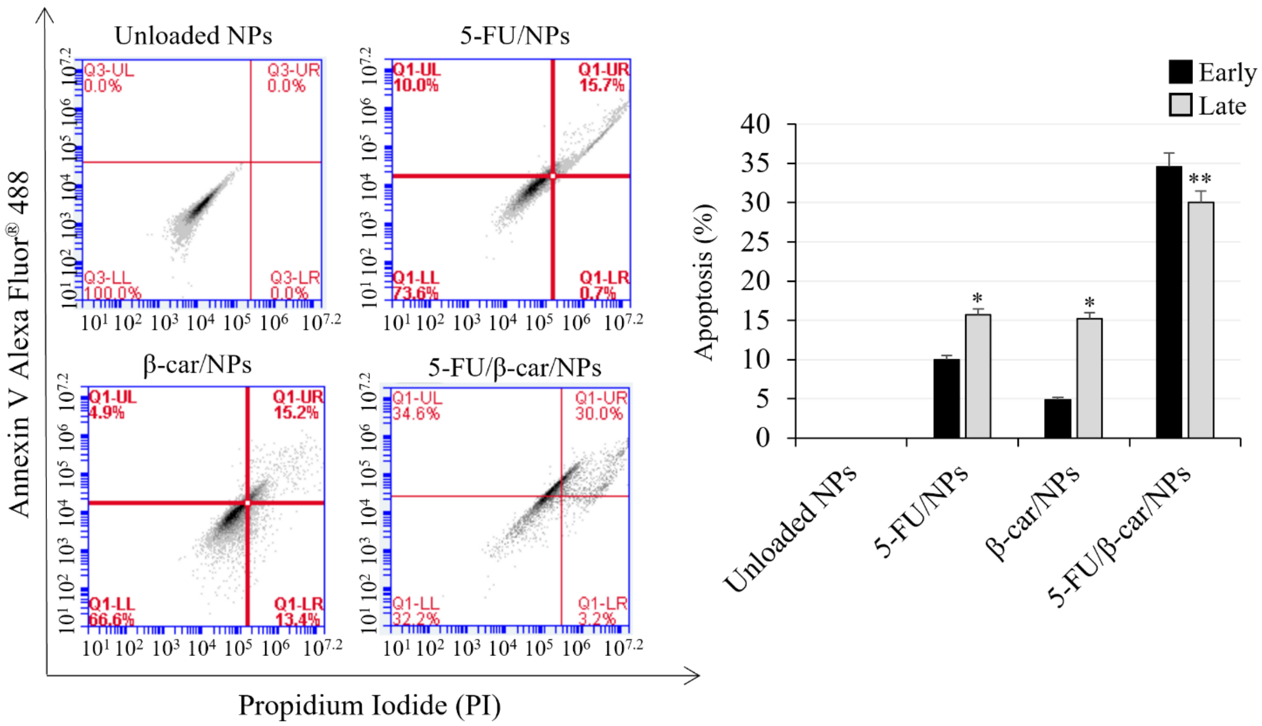

Figure S4. Effect of 5-FU and *β*-carotene treatment on apoptosis in HCT 116$^{p53-/-}$ cells. Representative flow cytometry dot blots with double Annexin V-Alexa Fluor 488/PI staining for cells treated with unloaded NPs, 5-FU/NPs, *β*-car/NPs, or 5-FU/*β*-car/NPs for 48 h. The bar diagram shows the percentage of early (Annexin V positive/PI negative) and late (Annexin V/PI positive) apoptotic cells. For each sample, at least 2 x 10^4 events were analyzed. *$p < 0.05$; **$p < 0.01$ vs unloaded NP-treated cells.

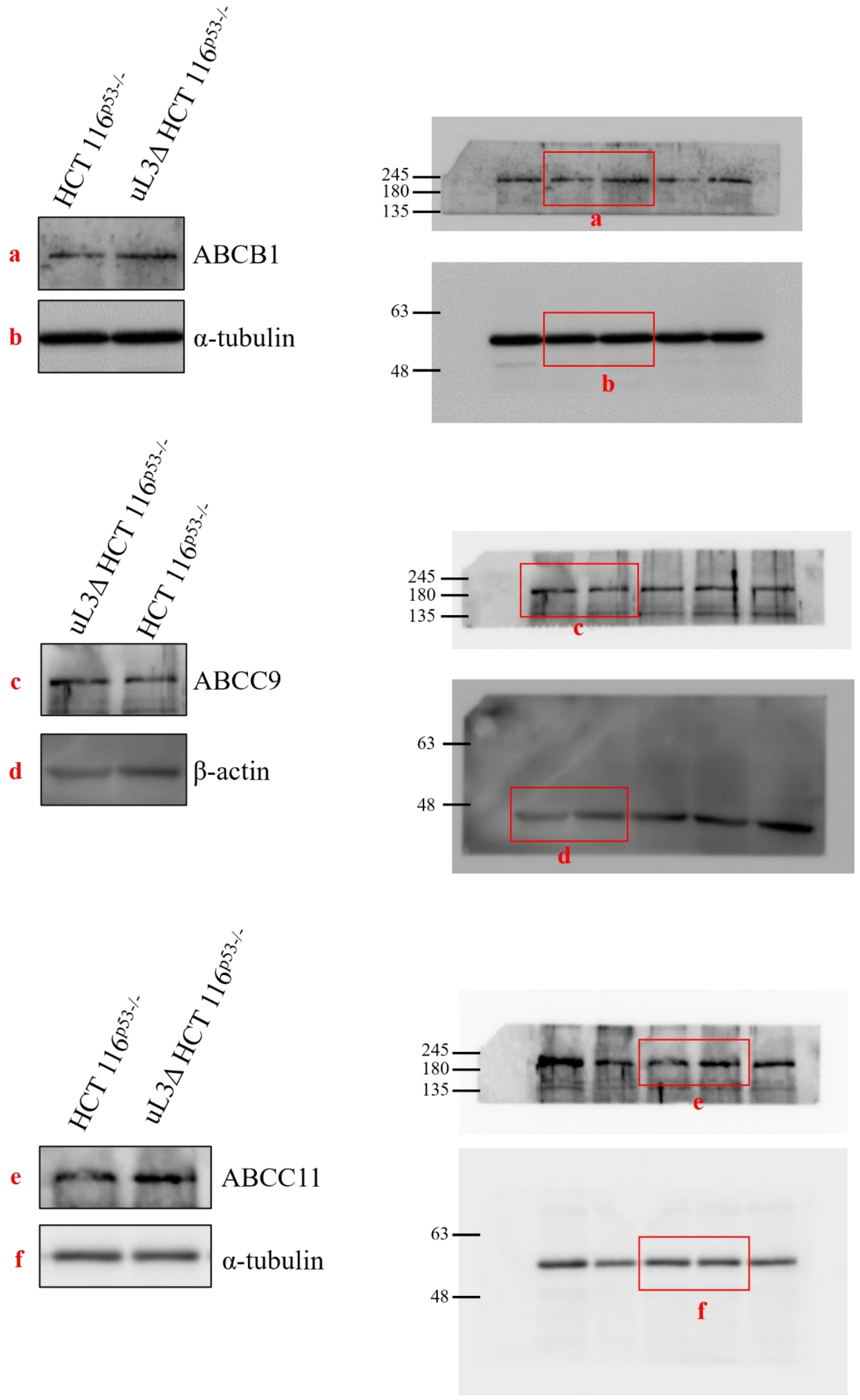

Figure S5. Full length blots of Figure S1.

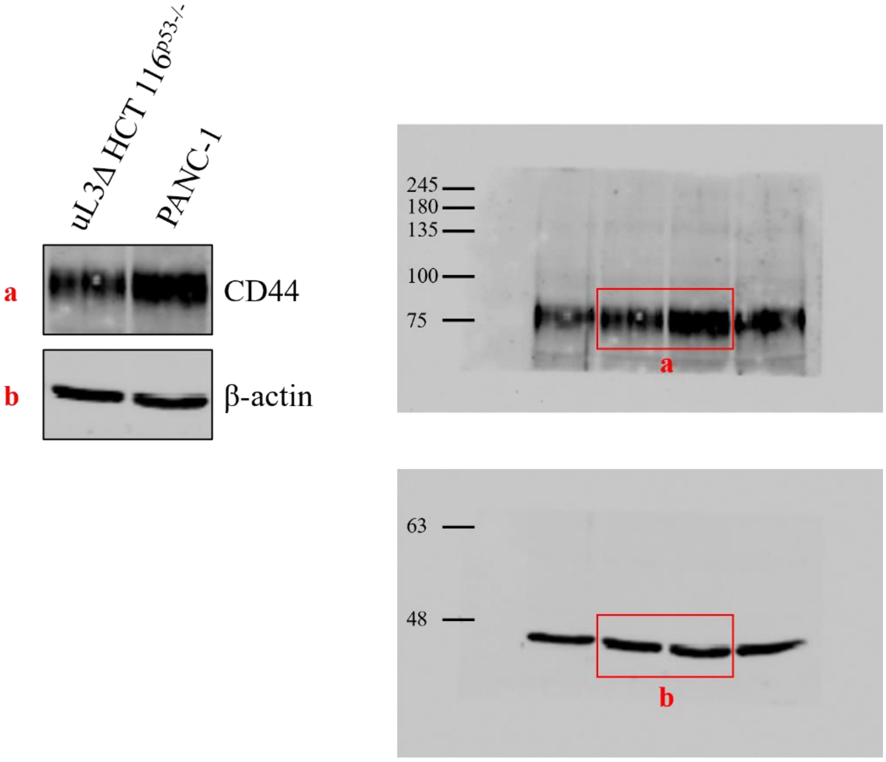

Figure S6. Full length blots of Figure S2a.

EXPERIMENTAL SECTION

Cell cultures

PANC-1 cell line (American Type Culture Collection, (ATCC) Manassas, Virginia, USA) were cultured in Dulbecco's Modified Eagle's Medium (DMEM) supplemented with 10% fetal bovine serum (FBS), 2 mM L-glutamine, penicillin-streptomycin 50 U/ml.

Western blot

Western blot analysis was performed as previously reported.[64] The membranes were challenged with anti-CD44 (Cell Signaling Technology, Danvers, MA, USA), anti-ABCC9, anti-ABCC11 (ThermoFisher, Loughborough, UK), and anti-ABCB1, anti-α-tubulin, anti-β-actin (Santa Cruz, Dallas, TX, USA). Proteins were visualized with an enhanced chemiluminescence detection reagent according to the manufacturer's instructions (Elabscience®, Houston, TX, USA).

Preparation of fluorescent NPs

Fluorescent NPs were prepared by solvent diffusion of an organic phase (2 mL) in an aqueous phase (4 mL of water with Pluronic F68 0.1%). The organic phase was prepared by dissolving 8 mg of PLGA 502H and 2 mg of PLGA-RHO (PLGA-Rhodamine B, Sigma-Aldrich, Italy) in 2 mL of acetone. After solvent removal under reduced pressure and room temperature, the sample was split into 4 Eppendorf tubes, centrifuged (5000 x g for 20 minutes) and redispersed in water (1 mL). After that, 125 µL of a PEI solution (2.5 mg/mL) was added, and the samples were washed by centrifugation (2800 x g for 20 min) and redispersed in 1 mL of water. Final NPs were obtained by adding 100 µL of HA in water (1 mg/mL). The interval between each addition was kept constant at 15 min. The hydrodynamic diameter (DH), polydispersity index, and zeta potential (ξ) of NPs were determined on a Zetasizer Nano ZS (Malvern Instruments Ltd). Results are reported as the mean of three separate measurements on three different batches ± standard deviation (n = 9).

Table S1. Properties of fluorescent NPs.

Batch	Size (nm ± SD)	P.I.	ζ (mV± SD)
RHO-PLGA NPs	224 ± 0.11	0.2	-26.3

Cellular uptake of NPs

The uptake of NPs was evaluated using Rhodamine B-labeled NPs (0.05 mg/mL) by measuring the incorporation of the fluorochrome Rhodamine B in uL3ΔHCT 116 $^{p53-/-}$ cells. Briefly, cells were plated at a density of 1×10^4 cells per well in 24-well plates and pretreated with free HA (10 mg/ml) for 1 h before the addition of NPs. Then, cells were incubated with 50 µg/mL of Rhodamine B-labeled NPs. After 2 h, cells were collected and analyzed by fluorimetry. The fluorescence was measured with a Cary Eclipse fluorescence spectrophotometer (Varian). Excitation and emission wavelengths were 546 and 568 nm, respectively.

CCK-8 assay

The CCK-8 kit (Dojindo, Shanghai, China) was used to measure cytotoxicity of 5-FU/NPs, *β*-car/NPs or 5-FU/*β*-car/NPs on uL3ΔHCT 116 $^{p53-/-}$ cells. A total of 5,000 cells in a volume of 100 µl per well were cultured in three replications in a 96-well plate. The CCK-8 was mixed with medium (10 µl per well) into each well and incubated at 37°C, 5% CO_2 for 120 min. The cell viability was determined by the measurement of the OD_{450} value at 48 h of treatment.
